# Efficacy of Interleukin 8 (IL-8) in the detection of urinary tract infection

**DOI:** 10.3389/fimmu.2026.1684579

**Published:** 2026-02-12

**Authors:** Jacqueline Barnett, Janice Kiely, Richard Luxton, Nicola Morris, Marcus Drake

**Affiliations:** 1 School of Applied Sciences, University of the West of England, Bristol, United Kingdom; 2 Institute of Bio-Sensing Technology, University of the West of England, Bristol, United Kingdom; 3 Bristol Urological Institute, North Bristol NHS Trust, Southmead Hospital, Bristol, United Kingdom; 4 Department of Surgery and Cancer, Imperial College London, London, United Kingdom

**Keywords:** cytokines, ELISA, IL-8, inflammatory markers, lactoferrin, urinary tract infection

## Abstract

**Introduction:**

Urinary tract infection (UTI) is the most common bacterial infection independent of age. Currently, the gold standard for diagnosis of UTI is based on the presence of bacteria together with white blood cells in the urine to distinguish significant infection vs contamination. Unfortunately, white blood cell count in the urine is non-specific. An ideal biomarker should be elevated in all UTIs, irrespective of the causative organism, and remain low in the absence of infection or systemic inflammation. The purpose of this proof-of- concept study was to examine whether IL-8 could act as an efficacious biomarker for UTI infection in combination with confirmed bacterial infection.

**Methods:**

In this study, IL-8 levels were measured in anonymised urine samples sent to the pathology laboratory for microbiology testing using commercially available ELISA kits. The urine samples tested had laboratory confirmed infection with *Escherichia coli, Proteus*, or *Klebsiella* bacteria.

**Results:**

The results demonstrated that although the IL-8 levels were variable for the 3 different causative agents of UTI tested, there was a significant difference between no growth samples and combined UTI samples. Of other biomarkers tested only lactoferrin gave comparable results to IL-8.

**Discussion:**

The IL-8 data presented is consistent with other published work and suggests that testing for IL-8 with measurement of uropathogenic bacteria could be a sensitive screening test for an accurate diagnosis of active UTI infection, excluding contamination and systemic inflammation. Larger studies with integrated clinical data are necessary to explore this further.

## Introduction

Recommendations from the US Food and Drug Administration (FDA) (1) and the European Medicines Agency (EMA) (2), defines urinary tract infection (UTI) as a combination of symptoms, the presence of white blood cells in the urine (pyuria), and the presence of bacteria in the urine (bacteriuria). Pyuria is considered important because it indicates inflammation, reducing the likelihood that bacteriuria represents mere colonisation or contamination. Nonetheless, definitions of UTI applied are surprisingly varied (3). Notably, a minority of studies required the presence of pyuria in their UTI definition, and furthermore there were variations in the threshold of pyuria concentration. A recent consensus proposed specific levels of pyuria into a reference standard, encouraging quantification of pyuria in studies done in all health-care settings (3).

Pyuria is non-specific, potentially reflecting sample contamination, or an inflammatory process due to a urinary tract or systemic condition (4). Hence, a more specific UTI biomarker than pyuria has the potential to improve UTI diagnosis (5). Interleukins (IL) are cytokines associated with immune response and may serve as biomarkers to indicate infection rather than colonisation. Elevated levels of specific cytokines, namely interleukins in urine and blood have been correlated with various infectious agents and inflammatory conditions, offering the potential for early detection and monitoring (4). Urinary IL-6 and IL-8 levels are increased during the acute phase of a UTI (6). Another study identified IL-8 as a predictor of inflammation associated with UTI (7), this is consistent with IL-8 being a potent neutrophil chemoattractant and activator. However, interleukins can also be elevated in urine as a consequence of systemic disease, such as rheumatoid arthritis and other inflammatory conditions. Such systemic conditions potentially can be associated with increased risk of UTI, as a result of the condition or any immuno-modulatory therapy given for the condition. This could restrict clinical utility of interleukin detection as a UTI biomarker by reducing specificity.

In this study, we have evaluated urine samples which had been split from samples sent to the pathology lab to be tested for UTI infection. Initially, we investigated the IL-8 concentration in samples that were positive and negative by culture, for three bacteria causing UTIs including *Escherichia coli* which is the causative agent of 70–85% of uncomplicated UTIs. Building on the results, we then broadened the analysis to investigate whether an additional biomarker will provide stronger indication across all UTI’s caused by three uropathogenic organisms *Escherichia coli*, *Klebsiella* also a causative agents of uncomplicated UTIs and *Proteus* which is associated with complicated UTIs (e.g., catheter-associated, recurrent infections).

## Materials and methods

### Test samples

All culture positive urine samples were obtained from samples sent to the Severn Pathology Department at Southmead Hospital, Bristol for detection of UTI. Samples were collected in primary care using sterile tubes and stored at +4 °C. The study was undertaken as part of the NIHR funded project II-LB-0417-20004, Rapid diagnosis of Urinary Tract Infection in Primary Healthcare.

In the initial phase of the study, the urine samples measured were culture positive for *Escherichia coli* (n=15), *Proteus* (n=15) or *Klebsiella pneumoniae* (n=15) and culture negative (n=15). The bacteria investigated were selected as they are the cause of 90% of UTIs.

Urine samples selected for test in the second phase of the study were culture positive for *Escherichia coli* (n=60), *Proteus mirabilis* (n=60) or no growth (n=60). Samples from “health” volunteers (n=60) were obtained from hospital staff (i.e. active volunteers, not under any clinical investigation).

In the third phase, four additional inflammatory biomarkers were measured in patient urine samples. These inflammatory biomarkers have also been reported in the literature to increase in concentration in the urine of UTI patients. These were inflammatory markers: IL-6 (8–10) Lactoferrin (11, 12) Uromodulin also known as Tamm–Horsfall protein (THP) (13, 14). and Procalcitonin (15, 16). Urine samples selected for test in this comparative study were culture positive for *E.coli* (n=5), *Proteus mirabilis* (n=5) and *Klebsiellapneumoniae* (n=5). Uninfected samples used in this comparative study were obtained from the Severn Vale Pathology Department at Southmead Hospital Bristol and were samples sent for detection of UTI but which were culture negative.

### Biomarker measurement

All enzyme-linked immunosorbent assays (ELISAs) used in this study were obtained from commercial suppliers. These include Human IL-8/CXCL8 R&D Systems, Quantikine ELISA Kit, Catalogue number, D8000C; Human IL-8 ELISA Kit, Invitrogen Novex Catalogue number, KHC0081; Uromodulin (UMOD), (Tamm-Horsfall Protein) Human ELISA kit, Invitrogen, Catalogue number EHUMOD; Human IL-6 R&D Systems, Quantikine ELISA Kit, Catalogue number QK206; Human Procalcitonin ELISA kit, Invitrogen, Catalogue number EHPCT and Human Lactoferrin ELISA Kit, Invitrogen, Catalogue number EH309RB.

All assays were solid-phase sandwich ELISAs measuring target concentration using matched antibody pairs. Target-specific antibody pre-coated in the wells of the supplied microplate was used with samples, standards, or controls. The application of an enzyme labelled second antibody and substrate solution creates a coloured product with an intensity proportional to the biomarker concentration.

Urine samples were centrifuged at 2000rpm to remove debris. Where urine was not specifically mentioned in the ELISA kit instructions, the volume used in the assay was the same as that specified for other biological samples, e.g. serum. All assays were carried out as described in the manufacturer’s instructions. Briefly, for each ELISA, all reagents and samples were brought to room temperature before use. All reagents, standard dilutions, and samples were prepared as directed in the product insert. 100 µL of assay diluent was added to each well, followed by 50 µL of a range of concentrations of standard, control, or sample to each well. The plate was covered with a plate sealer and incubated at room temperature for 2 hours.

After incubation the plate sealer was removed, and the plate washed 4 times with wash buffer. Any residual buffer was removed by inverting the plate onto paper towels, this was then followed by the addition of 100 µL of antibody conjugate to each well. The plate was then covered with a plate sealer and incubated at room temperature for 1 hour. Following this, the plate sealer was removed, and the plate washed 4 times with wash buffer. Any residual buffer was removed by inverting the plate onto paper towels. 200 µL of substrate solution was then added to each well and the plate incubated at room temperature for 30 minutes in the dark. Colour development was then stopped by the addition of 50 µL of stop solution to each well. The plate was then read on a microtiter plate reader at 450 nm within 30 minutes. Sample values were determined from standard curves and expressed as pg/ml to allow direct comparison of concentrations.

Statistical analysis was performed using the Wilcoxon rank sum test with continuity correction and with significance defined as p<0.05. Statistical analyses were performed using R Statistical Software (v3.6.0; R Core Team 2019).

## Results

### Initial phase of the study

**Figure 1:**
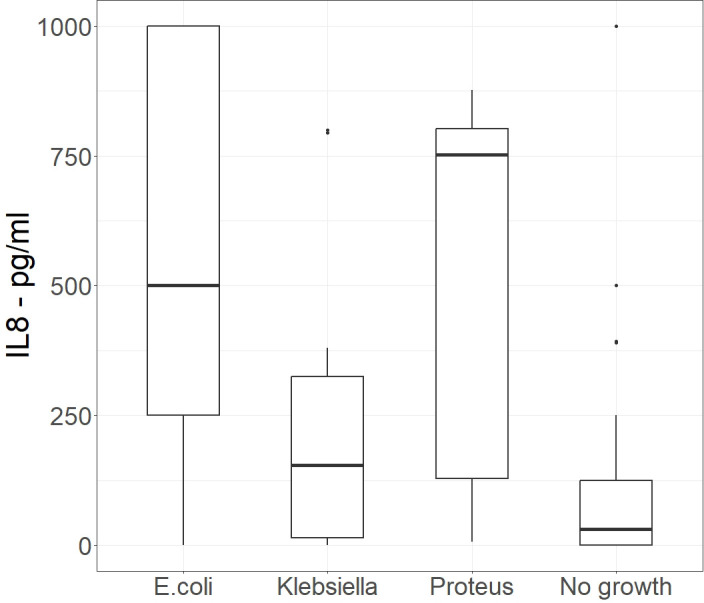
Initial study - Box and whisker plot of IL-8 concentrations present in urine samples that were culture positive for *E.Coli, Klebsiella and Proteus* and culture negative pathology samples, (n=15). Alt Text. **Figure 1**. Amount of Interleukin 8 in urine from samples which also grew specific bacteria in comparison to urine samples that did not grow bacteria.

In the initial small pilot study, 4 groups each consisting of 15 patient samples, were compared. The samples were from patients suffering from *E. coli*, *Proteus mirabilis*, or *Klebsiella pneumoniae* UTIs and a control group consisting of urine samples that showed no growth under the conditions tested. IL-8 measurements were performed on all the samples. **Table 1** shows the mean, median, and standard deviation of IL-8 measured in samples from patients with UTIs caused by *E. coli*, *Proteus mirabilis*, or *Klebsiella pneumoniae*, as identified in the pathology laboratory. **Figure 1** is a box and whisker plot showing the distribution of these results.

**Table 1:** Mean, median, and standard deviation of IL-8 concentrations in urine samples from the pathology lab, culture positive for *E.coli, Klebsiella* or *Proteus* or showing no bacterial growth (n=15 in each group).

Infecting organism	Concentration of IL-8 in urine (pg/ml)		
	Mean	Median	Std Dev
*E.coli*	486.3	500.0	364.66
*Klebsiella*	194.3	153.8	215.93
*Proteus*	521.5	752.1	341.93
No growth	132.2	31.3	229.07

There was no significant difference in IL-8 levels between samples with no bacterial growth and those with a *Klebsiella pneumoniae* UTI (p=0.1297). However, there was a significant difference between samples with no growth and those with *E. coli* (p<0.001) or *Proteus mirabilis* (p<0.0001) UTIs. Despite the mean and median values for patients with *Proteus mirabilis* UTI being higher than those with an *E. coli* infection, the difference in the IL-8 concentrations in the two groups was not significant (p=0.9123). Some culture-negative samples showed mildly elevated IL-8, likely reflecting an underlying inflammatory process. As the difference between *Klebsiella pneumoniae* and the go growth samples was not significant, the *Klebsiella pneumoniae* samples were not included in the Phase 2 of the study.

### Phase 2 – Extended study

**Figure 2:**
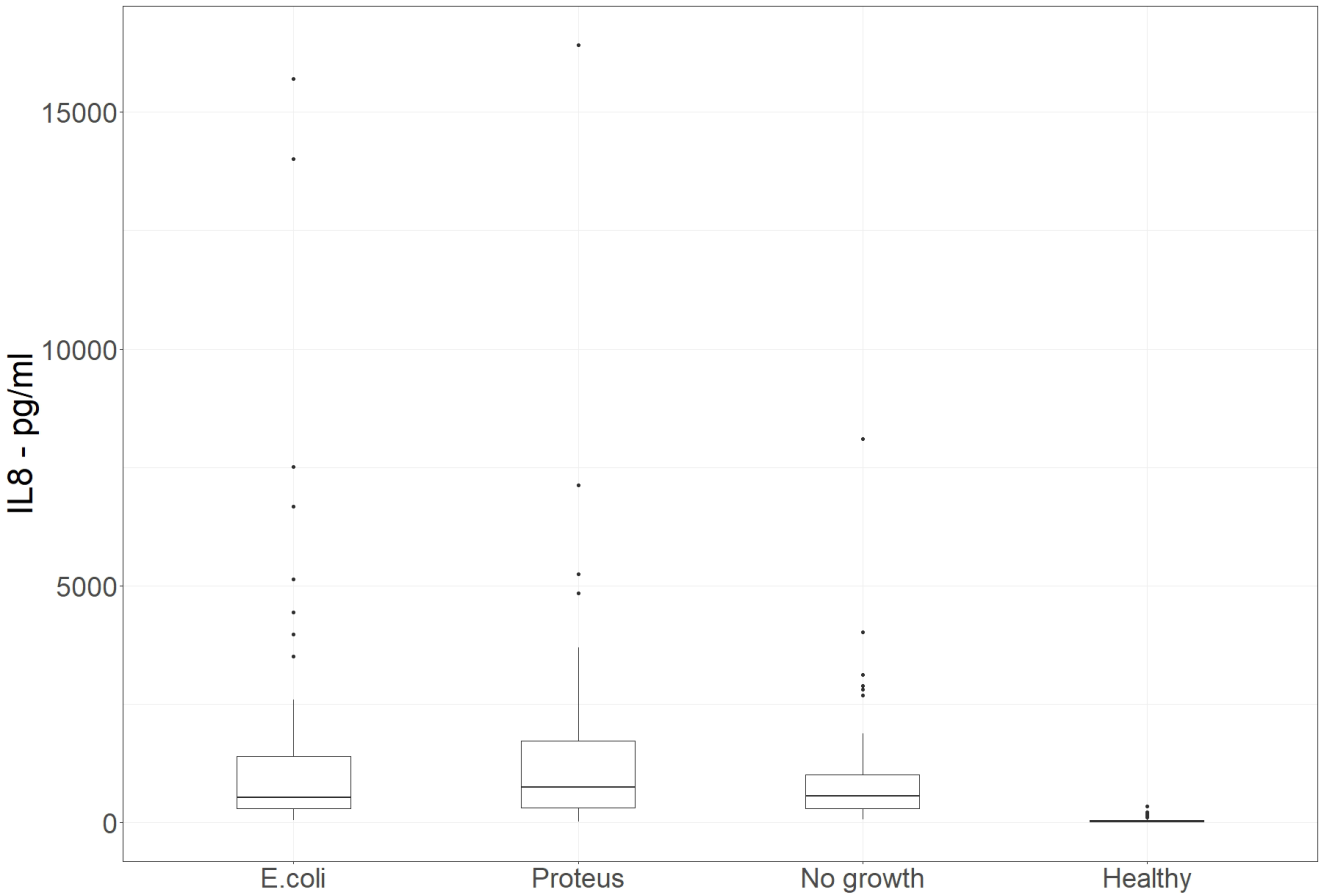
Phase 2 – Extended study. Box and whisker plot of IL-8 concentrations present in urine samples that were culture positive for *E. coli* or *Proteus*, pathology samples showing no bacterial growth, and healthy volunteers. (n=60 in each group). Alt Text. **Figure 2**. Range of concentration of Interleukin 8 in urine from samples in which specific bacteria grew in comparison to urine samples that did not grow bacteria and urine samples from healthy volunteers.

240 patient urine samples were evaluated. These were divided into 4 groups: one group of patients with an *E. coli* UTI, a second group of patients suffering from a *Proteus mirabilis* UTI, a third group consisting of the pathology controls that showed no growth, and a fourth group of urine samples from healthy volunteers. There were 60 samples in each group. **Table 2** shows the mean, median, and standard deviation of IL-8 concentration measured in the samples. **Figure 2** is a box and whisker plot showing the distribution of these results.

**Table 2:** Mean, median, and standard deviation of IL-8 concentrations in urine samples from the pathology lab, culture positive for *E. coli* or *Proteus*, or showing no bacterial growth, or health volunteers. (n=60 in each group).

Infecting organism	Concentration of IL-8 in urine (pg/ml)		
	Mean	Median	Std Dev
*E.coli*	1625	522	2916
*Proteus*	1523	748	2418
No growth	946	564	1249
Health volunteer	45.5	28.4	59

In this larger study, some patients had far higher concentrations of IL-8 detected in their urine compared to the values seen in the initial study. This is reflected in the higher mean values in the *E. coli* and *Proteus mirabilis* UTI groups, but the corresponding median values were similar to those observed in the initial study.

Despite a significant difference in the mean IL-8 concentrations between the no growth group and the groups with *E. coli* UTIs (p=0.0203) or *Proteus mirabilis* UTIs (p=0.0214), there was no significant difference in the overlap of the spread of results, as evaluated by the Wilcoxon rank-sum test with continuity correction (p=0.5116 for *E. coli* UTI and p=0.1096 for *Proteus mirabilis* UTI).

**Figure 3:**
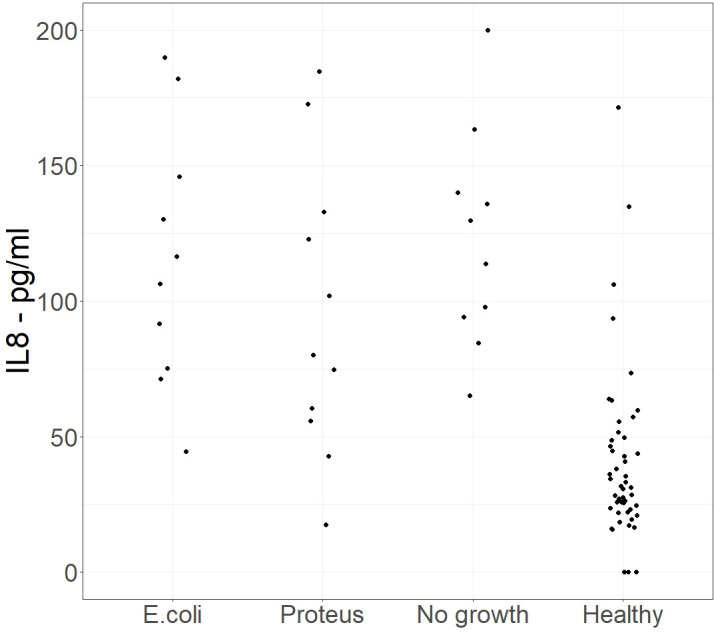
Phase 2 – Extended study. A jitter plot of samples with a low concentration of IL-8 concentration between 0 and 200 pg/ml for each of the 4 groups in the phase 2 study. Alt Text. **Figure 3**. Comparison of Interleukin 8 levels at low concentrations in urine samples which had bacterial growth or no growth.

**Figure 3** shows a jitter plot of the IL-8 concentrations in the range 0 to 200 pg/ml for each of the four groups. This highlighted the differences between the groups at low concentrations of IL- 8.

Measuring efficiency is a method of optimising the cut off value by combining the inversely proportional measures of sensitivity and specificity. Efficiency is the proportion of true positives and true negatives in the total populations at a particular cut off value. For our populations, the most efficient cut off value for IL-8 was 75 pg/ml (efficiency=0.92). This has a sensitivity of 0.955 and a specificity of 0.883. The sensitivity of IL-8 for *E. coli* UTI specifically was 0.9667 and 0.9167 for *Proteus mirabilis* UTI.

We assessed the clinical utility of IL-8 by identifying the threshold concentration that best discriminated infected from non-infected samples. Using the threshold value of 75 pg/ml, 88% of the samples from healthy volunteers were below 75 pg/ml, whereas for the samples from patients with laboratory confirmed UTIs only 5% of those with an *E.coli* infection and 6.7% with *Proteus mirabilis* infections had IL-8 concentrations below 75 pg/ml. This demonstrates a significant difference between IL-8 concentrations seen in samples from healthy volunteers compared with the IL8 concentrations from samples with *E.coli* or *Proteus* mirabilisUTI, or samples sent for investigation that showed no growth (Chi-squared=124.6, p <0.0001).

Following these observations, a further small study was performed to evaluate other biomarkers and explore the potential for combining markers to increase sensitivity or specificity.

### Phase 3 - Comparative study of biomarkers

In the third phase of this study additional biomarkers that have been associated with urinary tract infections in the literature were compared. The biomarkers tested were IL-8, IL-6, lactoferrin, procalcitonin and uromodulin (8–16). Twenty urine samples were selected from the pathology laboratory and divided into 4 groups. Three of the groups contained samples from patients with an identified UTI (*E. coli*, *Proteus mirabilis*, *Klebsiella pneumoniae*) and the fourth group contained samples that had no growth. **Table 3** shows the mean, median and standard deviation for each biomarker concentration in each group.

**Table 3:** Mean, median, and standard deviation of the concentration of multiple biomarkers in urine samples from the pathology lab culture positive for *E.coli, Klebsiella* or *Proteus*, or showing no bacterial growth. (n=60 in each group).

UTI	Biomarker	Concentration of Marker in urine (pg/ml)		
		Mean	Median	SDev
*E.coli*	IL6	44.7	0.802	95
*E.coli*	IL-8	751	314	829
*E.coli*	lactoferrin	6613	1306	12733
*E.coli*	procalcitonin	452	148	570
*E.coli*	uromodulin	1622	1413	479
*Klebsiella*	IL6	98.7	23.7	134
*Klebsiella*	IL-8	1494	1466	1189
*Klebsiella*	lactoferrin	10276	4833	16284
*Klebsiella*	procalcitonin	572	332	678
*Klebsiella*	uromodulin	3373	3519	1028
*Proteus*	IL6	88.1	27.1	121
*Proteus*	IL-8	1740	826	1557
*Proteus*	lactoferrin	11974	5789	11943
*Proteus*	procalcitonin	237	126	285
*Proteus*	uromodulin	3553	3079	1621
no growth	IL6	1.56	1.85	1.49
no growth	IL-8	86.1	0	188
no growth	lactoferrin	41	29.8	42.1
no growth	procalcitonin	851	480	680
no growth	uromodulin	3226	2851	980

**Figure 4:**
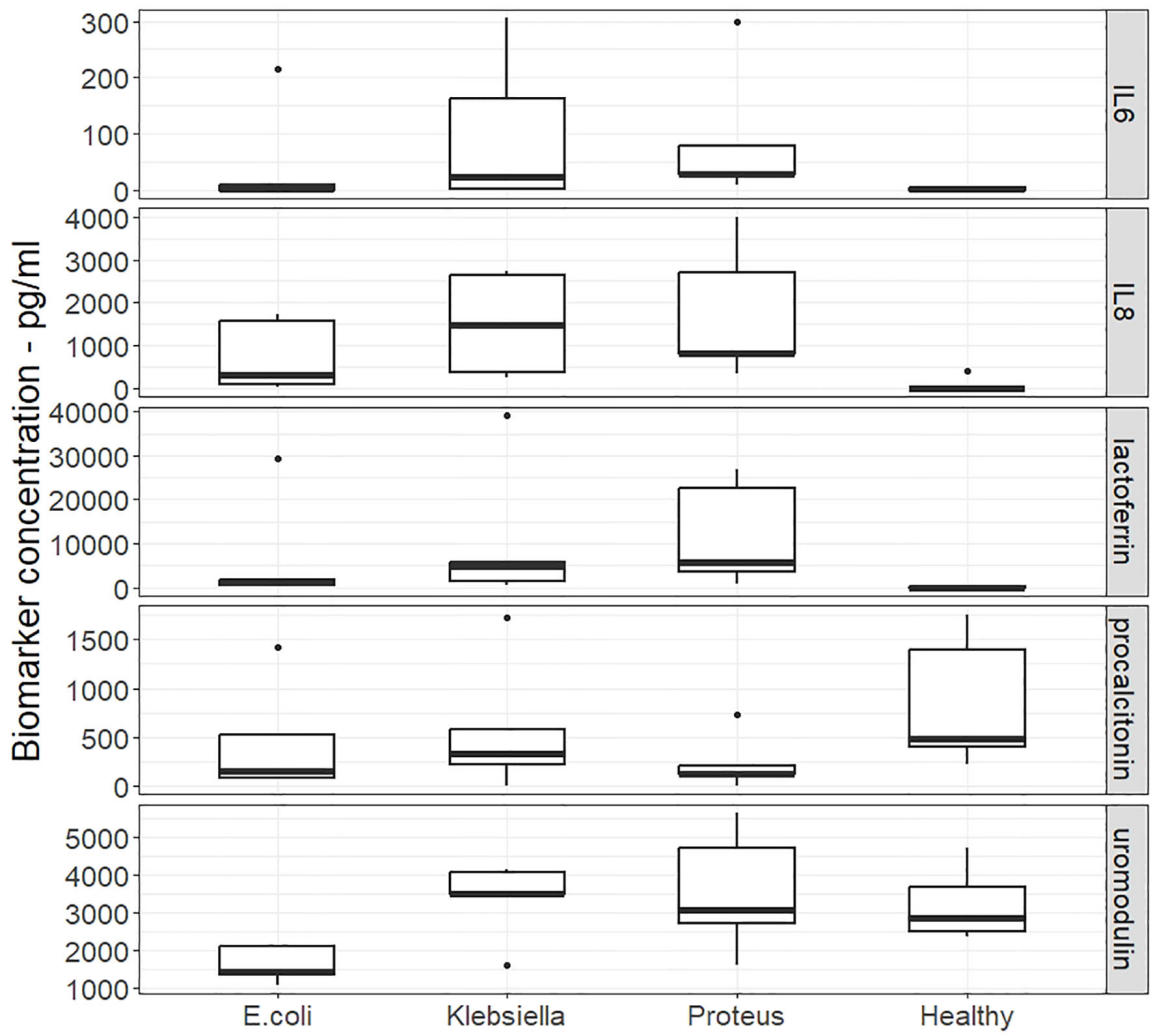
Phase 3 - Comparative study of biomarkers. Box and whisker plots of the concentrations of five biomarkers in urine samples that were culture positive for *E.Coli, Klebsiella* and *Proteus* and culture negative pathology samples Alt Text. **Figure 4**. Concentration of five biomarkers in urine samples which also grew specific bacteria in comparison to urine samples where bacteria did not grow.

A comparative analysis of the results highlighted the differential expression of the five biomarkers in various types of UTIs compared to no growth samples. IL-8 and lactoferrin emerge as promising candidates for distinguishing UTIs and warrant further research to validate these findings and explore the clinical implications for UTI diagnosis and management. **Figure 4** shows a Box and whisker plot for the different biomarkers across the different sample groups.

One of the key findings of this study is the significant difference observed in the concentrations of IL-8 and lactoferrin between no growth samples and combined UTI samples. IL-8 and lactoferrin exhibited significant differences with p-values of 0.0067 and 0.0005 respectively. When evaluating the individual causes of UTI in distinguishing from no growth samples, in the case of *E. coli* UTI lactoferrin showed the most significant difference in the concentrations of the biomarker (p=0.0317), for IL-8 the differences were just outside the significance threshold of 0.05 (p=0.0570). Uromodulin showed a significant reduction in concentration for the UTI samples compared with the no growth samples (p=0.0079). Samples from patients with a *Proteus mirabilis* UTI had the greatest significant difference from the no growth samples for IL-8 (p=0.0020), with lactoferrin also having a significant difference in concentration (p=0.0079). Samples growing *Klebsiella pneumoniae* had significant differences from the no growth samples for IL-8 (p=0.0344) and lactoferrin (p=0.0079). None of the other biomarkers showed a significant difference between the no growth and UTI groups. The drop in concentrations of procalcitonin in the UTI groups were nearly significant relative to no growth samples (p=0.0556).

Overall, the two biomarkers that exhibited the greatest discrimination were IL-8 and lactoferrin, reflected in the p values, but the magnitude of the changes are evidenced by the median values. For IL-8, the increase in median concentration is in the order of 100s of picograms per millilitre; *E. coli* 314 pg/ml, *Proteus mirabilis* 826 pg/ml, *Klebsiella pneumoniae*1466 pg/ml. In comparison, the lactoferrin median values increased in the order of 1000s of picograms per millilitre; *E.coli* 1306 pg/ml, *Proteus mirabilis* 5789 pg/ml, *Klebsiella pneumoniae* 4833 pg/ml. Despite this difference in magnitude and the significant difference from no growth samples, there was not a significant difference between IL-8 and lactoferrin concentrations recorded in each of the UTI groups. Thus, measuring both lactoferrin and IL-8 may not offer any significant benefit.

Of the other biomarkers tested, only uromodulin demonstrated any significant difference in concentration from the no growth sample group and that was for samples from patients with an *E. coli* UTI. Procalcitonin and IL6 did not show significant results in this small study. Although not significant, IL6 appeared to be raised more in samples from *Klebsiella pneumoniae* and *Proteus mirabilis* UTIs compared with *E. coli* UTI.

## Discussion

From these results IL-8 is elevated in patients with UTI caused by *E. coli* and *Proteus mirabilis*. In phase 1 of the study, the elevation of IL-8 seen in the *Klebsiella pneumoniae* UTIs was not significant over the IL-8 concentrations seen in the no-growth samples. The no growth samples, sent to the pathology lab for testing, had IL-8 concentration significantly greater than the concentrations seen in the healthy volunteer group. This suggests that an unexplained inflammatory response is occurring in ‘no-growth’ patients.

In fact, in the larger scale, phase 2 of the study, there was no significant difference in the IL-8 concentration from the no growth samples compared with those with an *E.coli* or *Proteus mirabilis*UTI samples. The high concentrations of IL-8 in the no growth controls suggest that other unexplained inflammatory conditions are being detected. This may be due to the presence of other bacteria that are not culturable under standard conditions and potentially responsible for UTI symptoms. However, when compared with the group of healthy volunteers, the IL-8 concentrations in all the groups were significantly higher (p<0.0001), which reinforces the explanation that the elevated IL-8 concentration in the no growth samples did reflect an unexplained inflammatory response.

The clinical utility of IL-8 in differentiating *E.coli* and *Proteus mirabilis* UTI from healthy urine samples was high with a sensitivity of 0.955 and 0.9667 and a specificity of 0.883 and 0.9167 for *E.coli* and *Proteus* mirabilis respectively. Only five patients with *Proteus mirabilis* UTIs and 2 patients with *E. coli* UTIs had IL-8 values less than 75 pg/ml. Overall, a minority of patients (5.8%) with an identified UTI may not have an elevated IL-8 concentration. On the other hand, 7/60 healthy volunteers had IL-8 concentrations greater than 75 pg/ml, four of which were slightly raised and 3 had concentration greater that 250 pg/ml. This suggests that even in “healthy” volunteers the raised IL-8 is in response to an unexplained, sub-clinical inflammatory response which in the normal course of day-to-day life would never be identified.

In phase 3, we saw that different biomarkers were elevated in different proportions in the UTI’s caused by three different bacterial species. The strongest was the increase in lactoferrin concentration - in terms of its absolute and percentage increase. The other significant observation was reduction of uromodulin in *E. coli* UTIs compared with no growth samples. Of the samples in the no growth group, 3 had undetectable IL-8 and the other had a concentration of 422 pg/ml, suggesting that 3 of the patients did not have an inflammatory response but the other patient may have had an underlying unexplained inflammatory response.

The markers tested are not exhaustive; additional biomarkers, such as calprotectin for paediatric UTIs, may be relevant (17, 18). These initial studies have indicated that IL-8 and Lactoferrin could be useful in confirming active urinary tract infection. In addition growth of uropathogenic bacteria without an increase in IL-8/Lactoferrin/inflammatory markers would indicate that the bacteria are present due to contamination. Samples with a large increase in inflammatory markers that are not culture positive may suggest further investigation is necessary. Not all bacteria that cause UTIs can be cultured, and DNA sequencing may be necessary to identify all organisms present.

This study was performed to provide preliminary proof-of-concept information about the value of measuring IL-8 and/or other inflammatory markers to improve the diagnosis of UTI, where specific UTI causing bacteria are confirmed to be present, the initial results are promising, The current study correlated biomarker findings with laboratory results and had very limited clinical information since anonymised samples were studied retrospectively. There are important features relating to clinical presentation, risk factors and past medical history which are important for developing the initial findings from the current study, and which will need a prospective clinical utility study to explore further.

## Conclusion

Urinary IL-8 could be used as a screening test for urinary tract infections with a high sensitivity (0.96). In some cases, the test would also detect an inflammatory response even when no bacteria were cultured from the urine, which might warrant further clinical investigation. Lactoferrin is another possible candidate as a biomarker in a rapid screening test giving good discrimination (p=0.0079) from non-UTI patients (defined by culture). Using a rapid testing platform, such as offered by lateral flow or biosensor technologies, a urinary test for IL-8 and/or Lactoferrin or both could serve as a rapid confirmation of the presence of UTI-related inflammation in uncomplicated adult UTI. Additional validated inflammatory markers may be required to test additional specific patient groups. The integration of rapid tests for biomarkers, such as IL-8, with rapid culture-free methods for bacterial identification and quantification such as that described by (19) could enable the prompt initiation of therapy and reduce the incorrect prescribing of antibiotics.

## Data Availability

The raw data supporting the conclusions of this article will be made available by the authors, without undue reservation.
